# Quantitative trait locus mapping and improved resistance to sclerotinia stem rot in a backbone parent of rapeseed (*Brassica napus* L.)

**DOI:** 10.3389/fpls.2022.1056206

**Published:** 2022-11-10

**Authors:** Xiaohui Zhang, Xiang Li, Huining Li, Zhuanrong Wang, Rui Xia, Jin Hu, Pengfei Wang, Xianming Zhou, Lili Wan, Dengfeng Hong, Guangsheng Yang

**Affiliations:** ^1^ National Key Laboratory of Crop Genetic Improvement, Huazhong Agricultural University, Wuhan, China; ^2^ Sanya Nanfan Research Institute of Hainan University, Hainan Yazhou Bay Seed Laboratory, Sanya, China; ^3^ College of Tropical Crops, Hainan University, Haikou, China; ^4^ Institute of Crops, Wuhan Academy of Agricultural Sciences, Wuhan, China

**Keywords:** quantitative trait locus mapping, sclerotinia stem rot, flowering time, backbone parent, rapeseed

## Abstract

There are three main challenges to improving sclerotinia stem rot (SSR) resistance in rapeseed (*Brassica napus* L.). First, breeding materials such as the backbone parents have not been extensively investigated, making the findings of previous studies difficult to directly implement. Second, SSR resistance and flowering time (FT) loci are typically linked; thus, use of these loci requires sacrifice of the rapeseed growth period. Third, the SSR resistance loci in susceptible materials are often neglected, thereby reducing the richness of resistant resources. This study was conducted to investigate the stem resistance, disease index, and FT of a doubled haploid population consisting of 151 lines constructed from the backbone parent 19514A and conventional rapeseed cultivar ZY50 within multiple environments. Quantitative trait locus (QTL) mapping revealed 13 stem resistance QTLs, 9 disease index QTLs, and 20 FT QTLs. QTL meta-analysis showed that *uqA04*, *uqC03.1*, and *uqC03.2* were repeatable SSR resistance QTLs derived from different parents but not affected by the FT. Based on these three QTLs, we proposed a strategy for improving the SSR resistance of 19514A and ZY50. This study improves the understanding of the resistance to rapeseed SSR and genetic basis of FT and demonstrates that SSR resistance QTLs can be mined from parents with a minimal resistance level difference, thereby supporting the application of backbone parents in related research and resistance improvement.

## Introduction

Rapeseed (*Brassica napus* L.) is among the most important oil crops worldwide and its supply affects the stability of edible oil production. The yield of rapeseed is affected by several factors, such as yield potential, flowering time (FT), and yield stability. Poor yield stability is the greatest contributor to low yields. Sclerotinia stem rot (SSR) is a fatal disease in plants and is caused by the typical dead trophic pathogen *Sclerotinia sclerotiorum*. This disease is prevalent during rapeseed growth and can cause serious yield losses (Bolton et al., 2006). In China, SSR occurs in all rapeseed planting areas, particularly in areas where winter rapeseed is grown, resulting in related annual yield losses of 10–20%. In areas severely affected by SSR, yield loss can exceed 80% or lead to complete harvest loss (Xu et al., 2014; Yu et al., 2020; Ding et al., 2021). SSR can also reduce the oil content of rapeseed seeds and affect the quality of rapeseed oil, which may negatively impact human health (Pressete et al., 2019). Compared with chemical prevention and control, improving the SRR resistance and breeding resistant cultivars of rapeseed are more economical, efficient, and sustainable methods for mitigating damage caused by SSR (Ding et al., 2021).

The basic requirements for SSR resistance breeding are to establish efficient and accurate identification methods and search for resistant germplasm resources. Various methods for SSR resistance identification have been proposed and are widely used at the seedling, flowering termination, and mature stages; however, no germplasm resources with high resistance or complete immunity have been identified (Ding et al., 2021). Although a series of genes was confirmed *via* reverse genetics to be involved in regulating SSR resistance (Wang et al., 2014; Wang et al., 2019a; Jiang et al., 2020; Wang et al., 2020; Cao et al., 2022; Zuo et al., 2022) and progress has been made in understanding its regulatory network (Cao et al., 2016; Wang et al., 2019b; Hu et al., 2021; Xu et al., 2021; Zhang et al., 2022), it is difficult to apply these results in the short term because of the limited material specificity and transgenic restriction policies. Thus, the main strategy used to improve SSR resistance is constructing isolated populations and analyzing the genetic basis of SSR resistance of two inbred lines showing a large discrepancy in their SSR resistance levels, followed by exploration and utilization of QTLs related to SSR resistance. Backbone parents with a high combining ability often produce excellent hybrid cultivars (Fradgley et al., 2019; Ma et al., 2019; Chen et al., 2021). SSR resistance of the backbone parents and improvements in their SSR resistance have been studied at the breeding level, and the results can be rapidly applied in breeding. However, in this strategy, a large difference in the SSR resistance level is the main criterion for selecting parents, and backbone breeding parents that have undergone natural selection and strict artificial selection are rarely used as susceptible parents because their SSR resistance levels are not low enough. Using this strategy, numerous researchers have applied various methods for detecting SSR to discover a large number of SSR resistance-related QTLs in different growth stages of rapeseed (Zhao et al., 2006; Yin et al., 2009; Mei et al., 2013; Wu et al., 2013; Li et al., 2015; Wei et al., 2016; Wu et al., 2016a; Shao et al., 2022). However, the correlation between the results of different identification methods was low, as was the repeatability of QTLs mined in different studies; therefore, few or no QTLs have been applied to improve resistance to SSR (Ding et al., 2021). Nevertheless, these studies repeatedly confirmed that SSR resistance is not controlled by a single major locus but rather by multiple micro loci, thus revealing the genetic pattern of SSR resistance. Moreover, studies demonstrated that even relatively susceptible parents can provide a source of resistance (Wu et al., 2013). Thus, breeding materials can be directly used to construct populations for exploring resistance loci as a potential strategy to improve the SSR resistance of the backbone parents regardless of resistance differences in the parents.

SSR resistance in rapeseed is related to the stem strength (Shao et al., 2022), lignin content (Cao et al., 2022), glucosinolate content (Zhao and Meng, 2008), FT (Wei et al., 2014; Wu et al., 2019; Zhang et al., 2019), and other traits (Feng et al., 2021), and their interactions should be monitored during the improvement of SSR resistance. A study of the FT and SSR resistance of 521 rapeseed inbred lines revealed a significant negative correlation between the two traits, demonstrating that they had the closest relationship. Inbred lines with early FT were more susceptible to SSR, whereas those with late FTs were more resistant to SSR (Zhang et al., 2019). Furthermore, several studies showed that QTLs for FT colocalized with those for SSR resistance, confirming the genetic linkage between the FT and SSR resistance loci (Wei et al., 2014; Wu et al., 2019; Zhang et al., 2019). The pleiotropic effect of a single gene is one reason for the linkage between the FT and SSR resistance loci in *Arabidopsis thaliana* (Kidd et al., 2009; Li et al., 2012; Lyons et al., 2013; Singh et al., 2013; Lai et al., 2014), but this has not been clearly demonstrated in rapeseed. FT has a comprehensive impact on rapeseed cultivars, and differences in the FT directly affect the adaptability of rapeseed cultivars to different planting areas as well as yield (Raman et al., 2019; Kaur et al., 2021). Rapeseed improvement requires shorter growth times and earlier flowering periods. Therefore, improving the SSR resistance of rapeseed should not be at the expense of FT. When analyzing the QTL for SSR resistance and using the results to guide improvements in breeding parents, the QTL for FT should be avoided.

In this study, the temperature-sensitive pol cytoplasmic male sterile line 19514A (a backbone parent) and conventional cultivar ZY50, along with their constructed doubled haploid (DH) population containing 151 lines, were used to examine the resistance to SSR using a stem resistance (SR) assay at the flowering termination stage and disease index (DI) at the mature stage for two consecutive years. The SR QTLs and DI QTLs were explored based on a high-density genetic linkage map. Additionally, the phenotypes of the FT of the parents and their DH populations were investigated in multiple environments, and the genetic basis of the differences in the FT of the DH population was analyzed. Through colocalization analysis of the QTL for SR, DI, and FT, a SSR resistance improvement scheme for 19514A and ZY50 was proposed. This study improves the understanding of the genetic basis of SSR resistance and FT in rapeseed and provides useful information for improving SSR resistance in backbone parents and SSR resistance of 19514A and ZY50.

## Materials and methods

### Plant material and growth conditions

19514A (named G120) is a temperature-sensitive pol cytoplasmic male sterile line. This semi-winter backbone sterile line has been formulated with many hybrid cultivars that are promoted in the market but shows a low SSR level. ZY50 (named as 9172) is a conventional semi-winter rapeseed cultivar with excellent performance. The DH population constructed with ZY50 and 19514A contains 151 lines, for which a high-density genetic linkage map containing 910 single-nucleotide polymorphism markers and 187 simple sequence repeats markers was previously constructed (Liu et al., 2020). All plant materials were sown in the field of Wuhan (WH), Jingzhou (JZ), or Zhangye (ZY) during normal growing seasons. Each row consisted of 10–12 plants, with distances of 20 cm between individuals and 25 cm between rows. Conventional field management was conducted according to local planting practices.

### Stem inoculation assay

The parents and their DH population were planted at the experimental base of Huazhong Agricultural University in WH for two consecutive years from 2015 to 2016; three rows of each material were planted. Inoculation was performed as described by Wang et al. (2018) and Wu et al. (2013). The *S. sclerotiorum* isolate SS-1 was maintained and cultured on potato dextrose agar (PDA, 25% potato, 2.5% dextrose and 1.5% agar, pH 5.8). The isolate was cultured more than two cycles prior to inoculation at 23°C in darkness. At the flowering termination stage of rapeseed, agar discs (8 mm in diameter) were excised from the edges of growing fungal colonies and up-ended into the lids of 1.5- or 2.0-mL centrifuge tubes. These tubes were affixed with plastic wrap onto rapeseed stems at 30 cm from the ground. Disease severity was assessed twice by measuring the lesion length per pathogen infection spot at 7 and 14 days post-infection (dpi) (17 dpi in 2015). For each DH line, 10 plants showing a consistent growth status were selected for inoculation, and the single plant in the middle row was preferentially selected to reduce the impact of marginal effects. In the collected data, the average value of the six-middle data of each family was used as the phenotype value.

### Natural infection experiment

The parents and their DH population were planted in JZ for two consecutive years from 2015 to 2016. The experiments were performed in a randomized block design with two replicates, and each line was planted in three rows to ensure that there were approximately 30 plants. As described by Wang et al. (2018), five agar discs (8 mm in diameter) containing active mycelia were added to 500 ml of liquid potato medium (25% potato and 2.5% dextrose, pH 5.8) and incubated at 200 rpm for 3 days at 23°C in the dark. The mycelia were then fully interrupted and diluted to 10 L with water, and then the 5% suspension of *S. sclerotiorum* hyphae was sprayed onto the rapeseed plants at the full-flowering stage to increase disease-causing stress. Before harvest, the incidence level of each plant was evaluated as described by Zhou et al. (1993). The DI of each plot was calculated as DI = 100 Σ(i × n_i_)/(N × k), where i is the disease severity score from 0 to 4, n_i_ is total number of plants in each score, N is total number of plants evaluated in each plot, and k is the highest score (here, k = 4).

### FT evaluation

The parents and their DH population were planted in ZY for two consecutive years from 2015 to 2016; JZ in 2016; and WH in 2015, 2016 and 2018. The experiments were conducted in a randomized block design with two replicates, and each replicate was planted in three rows. The phenotypic values of individual plants in each line from the sowing date to 50% flowering were recorded as the flowering date.

### Data statistics and analysis

Data collection and preliminary analysis were performed using Microsoft Excel 2016. One-way analysis of variance, correlation analysis, and graphical presentation of data were performed using GraphPad Prism 8 software (GraphPad, Inc., La Jolla, CA, USA). The phenotypic data generated in this study are shown in **Supplementary Data 1**.

### QTL mapping and meta-analysis

QTL analysis was performed *via* composite interval mapping using WinQTL cartographer 2.5 software (Zeng, 1994). The walk speed was set to 1 cM. The limit of detection (LOD) threshold for each trait was determined using permutation testing with 1000 repetitions. A QTL was declared when the LOD score was greater than the threshold value; LOD scores corresponding to P < 0.05 were used to identify significant QTLs. QTLs repeatedly detected in different environments and different trait were integrated into consensus QTLs through meta-analysis using BioMercator 2.1 software (Arcade et al., 2004).

## Results

### Phenotypic identification and QTL mapping for SR

**Figure 1:**
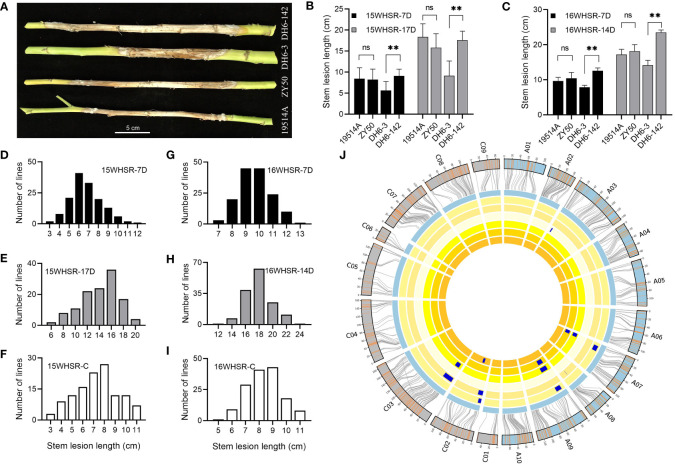
Phenotype and quantitative trait loci (QTL) mapping of stem resistance assay. **(A)** Disease lesions on the stems of the two parents and two double haploid (DH) lines at 14 days post-infection (dpi). Bar = 5 cm. **(B, C)** Stem lesion lengths of the two parents and two DH lines in 2015 and 2016. “-7D” indicates the data from the first measurement at 7 dpi and “-14D” and “-17D” indicate the data from the second measurement. Data are shown as the mean ± SD; ns indicates no significant difference, **P < 0.01 (one-way analysis of variance). **(D-I)** Distribution of stem lesion lengths of the DH population in 2015 **(D–F)** and 2016 **(G–I)**. “-C” indicates the difference between the two measurements. **(J)** Genetic linkage map and locations of QTLs for stem resistance (SR). From inside to outside, the six cycles represent 15WHSR-7D, 15WHSR-17D, 15WHSR-C, 16WHSR-7D, 16WHSR-14D, and 16WHSR-C. The two outermost cycles show a comparison of the linkage and physical maps of *Brassica napus*.

To identify the SR of the parents and their DH population, we performed stem inoculation experiments at maturity for two consecutive years. We recorded the phenotype data at 7 days after inoculation (15WHSR-7D and 16WHSR-7D) and several days after inoculation (15WHSR-17D and 16WHSR-14D). The difference between the two sets of data were used as the third group of phenotype data (15WHSR-C and 16WHSR-C). The results showed that the SR of 19514A and ZY50 was not significantly different at all four phenotype collections over two years; however, stable and significant differences in SR were observed between specific DH lines (**Figures 1A-C**). In the DH population, SR showed a continuous distribution in all six datasets, with the performance of the parents in the middle of that of the DH population (**Figures 1D–I**). In correlation analysis, the three sets of data in the same year showed significant positive correlations. Across different years, except for the 15WHSR-7D and 16WHSR-7D groups, the other two groups of data also showed significant positive correlations (**Supplementary Table 1**). This result confirms the reliability of the experimental data and suggests that multiple loci regulate SR in the DH population, and that both parents can provide resistance sources.

To mine the loci controlling SR in the DH population, we combined the existing high-density genetic linkage map and these six datasets for QTL mapping. We detected 13 QTLs with LOD values of 2.65–4.84 and phenotypic variation of 4.3–11.2%. The additive effects of different QTLs were from different parents, indicating that both parents provide a source of resistance (**Supplementary Table 2**). The identified QTLs were distributed on the A03, A07, A08, A09, C02, and C03 linkage groups, and the confidence intervals of QTLs in different datasets overlapped (**Figure 1J**). Among these QTLs, *qSRA07-1* and *qSRA09-1* were detected repeatedly in 2015, and *qSRC02-2* was detected repeatedly in 2016, showing that different data collection methods can identify stable QTLs. *qSRA09-1* and *qSRC03-1* were detected across different years and considered as stable QTLs, and thus should be further evaluated.

### Phenotypic identification and QTL mapping for DI

**Figure 2:**
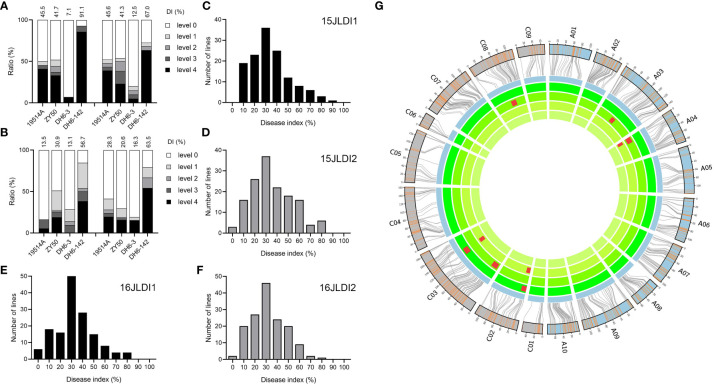
Phenotype and quantitative trait loci (QTL) mapping of natural infection experiment. **(A)** Disease indices (DIs) of the two parents and two double haploid (DH) lines in two replicates in 2015. The histograms represent the ratio of plants with different disease levels. **(B)** DIs of the two parental lines and two DH lines of two replicates in 2016. **(C–F)** Distribution of DIs of the DH population in 2015 **(C, D)** and 2016 **(E, F)**. **(G)** Genetic linkage map and locations of QTLs for DI. From inside to outside, the four cycles represent 15JZDI1, 15JZDI2, 16JZDI1, and 16JZDI2. The two outmost cycles show a comparison of the linkage and physical maps of *Brassica napus*.

To measure the resistance of the parents and their DH population to SSR using the DI, we investigated the phenotypes of four replicates over two years. As with SR, DI did not significantly differ between the two parents, although there were stable significant differences between specific DH lines (**Figures 2A, B**). The DI of the DH population showed a large range and an approximately normal distribution, indicating that multiple loci in the DH population simultaneously regulate the DI (**Figures 2C–F**). Correlation analysis revealed significant positive correlations among the four replicates (**Supplementary Table 3**), demonstrating the stability of this method for identifying disease resistance and the reliability of the data.

A total of nine QTLs were detected in the four replicates, with LOD values ranging from 2.54 to 5.82, explaining 5.3% to 12.2% of the phenotypic variation. The additive effects of these QTLs ranged from 3.61 to 6.42, with resistance derived from different parents (**Supplementary Table 4**). According to the confidence intervals, six QTLs were integrated into three consensus QTLs on chromosomes A04, C02, and C03 (**Figure 2G**). These three QTLs showed similar phenotypic variation and additive effects, and thus may jointly regulate SSR resistance at the same level.

### QTL mapping for FT

To mine the QTL regulating FT in the DH population, we investigated 10 replicates of FT in five environments. Correlation analysis showed significant positive correlations among the 10 replicates of FT data (**Supplementary Table 5**). In most environments, the FT of the DH population was distributed continuously over 20 days. In WH in 2015, the FT lasted for 30 days. None of the replicates showed an obvious Mendelian distribution, and most replicates did not conform to the standard normal distribution. These results indicate that the FT of the DH population is controlled by a major locus and multiple minor loci (**Supplementary Figure 1**).

**Figure 3:**
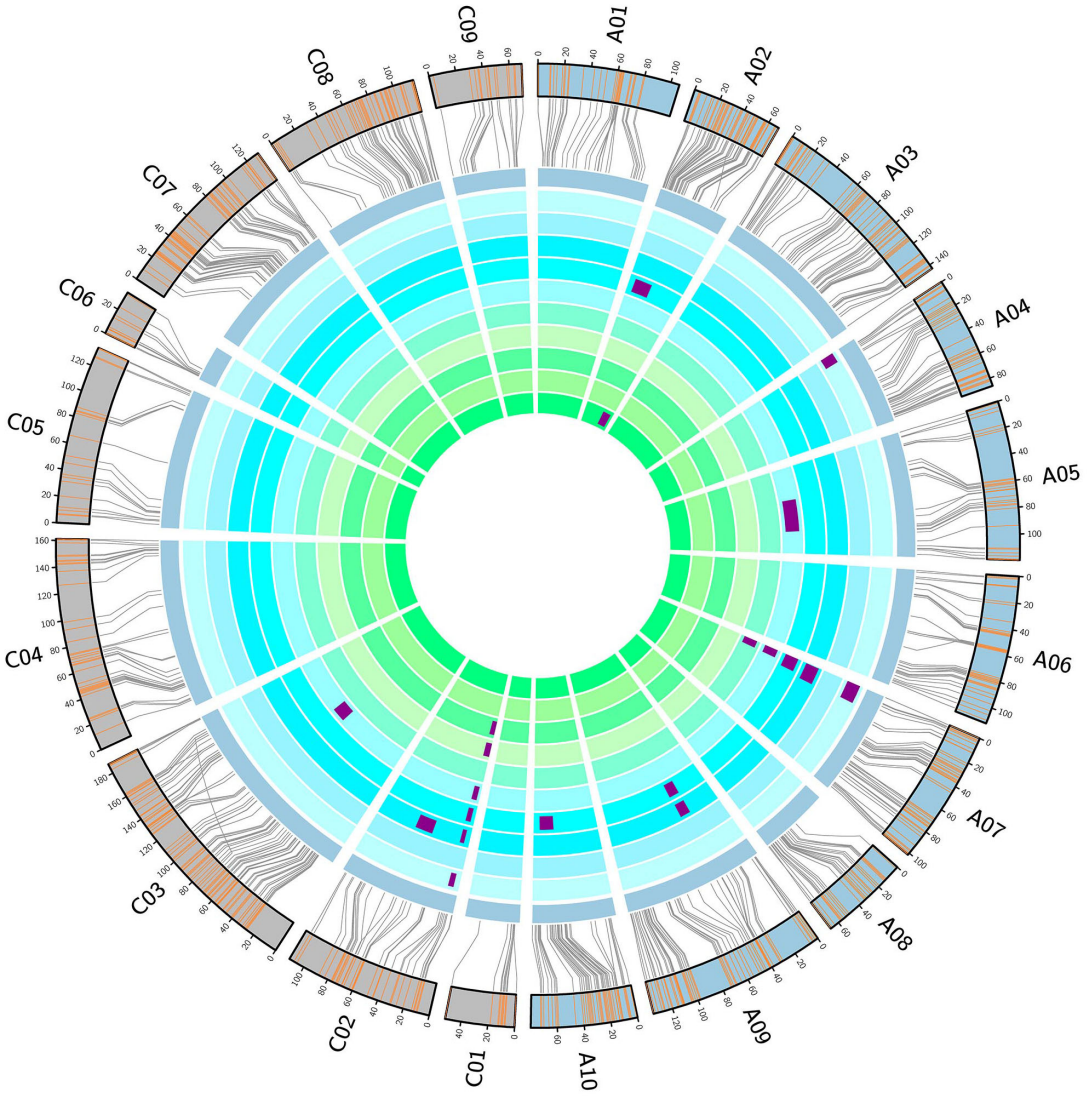
Genetic linkage map and locations of quantitative trait loci (QTL) for flowering time (FT). From inside to outside, the ten cycles represent 15ZYFT1, 15ZYFT2, 16ZYFT1, 16ZYFT2, 15WHFT1, 15WHFT2, 16WHFT1, 16WHFT2, 16JZFT1, and 16JZFT2. The two outmost cycles show a comparison of the linkage and physical maps of *Brassica napus*.

The results of QTL mapping showed that 20 QTLs for FT were detected in 8 of 10 replicates, among which 3 and 17 QTLs were detected in the spring and winter rapeseed growth region, respectively (**Figure 3**). These QTLs were distributed on chromosomes A02, A04, A07, A09, A10, C02, and C03, with *qFTC02-1* detected in a total of six repeats in the three locations with LOD values of 2.53–3.04, 26.03–27.07, and 15.39, phenotypic contribution rates of 6.2–8.1%, 43.8–48.2%, and 27.6%, and additive effects of 1.65d–1.85d, 3.48d–5.27d, and 2.44d. Another QTL, *qFTA07-1*, was detected in five replicates in WH and JZ, among which four replicates except for 15WHFT1 showed LOD values of 2.77–4.38, phenotypic contributions of 3.3–4.9%, and additive effects of 0.93d–1.32d. In addition, the QTL *qFTA9-1* was detected in two replicates in WH (**Supplementary Table 6**). The above results confirm that the FT differed within the DH population and was simultaneously regulated by multiple loci. In WH and JZ, *qFTC02-1* was a stable major locus, whereas *qFTA07-1* and *qFTA09-1* were stable minor loci.

### QTL meta-analysis for SR, DI, and FT

To analyze whether the previously detected QTLs for SSR resistance are affected by the FT, we performed a meta-analysis of all QTLs for the above three traits. The results showed that seven QTLs were repeatedly detected, among which *uqC02* was stably detected in all three traits and *uqA07* was detected in SR and FT. These results indicate that the two QTLs affect FT to influence the SSR resistance of rapeseed. *uqA09.1* and *uqA09.2* were repeatedly detected in SR and FT, respectively. Although they were identified as two different QTLs, their confidence intervals showed a small overlap; thus, we considered this QTL to play a role in SSR resistance while also affecting the FT. The remaining three QTLs, *uqA04*, *uqC03.1*, and *uqC03.2*, were stably detected in SR or DI and were not affected by the FT, although *uqA04* may be considered as a minor FT QTL in one replicate (**Table 1**). The above results indicate that the SSR resistance of this DH population was partly regulated by the FT. However, we identified several SSR resistance QTLs that were not related to the FT, which should be further evaluated.

**Table 1:** Unique quantitative trait loci (QTL) information in this study.

Name	Chr	Position (cM)	CI (cM)	SR	DI	FT	References
uqA04	A04	4.19	0.47-7.91		15JZDI2/16JZDI1	16JZFT2	
uqA07	A07	2.65	0-5.54	15WH-17D/15WH-C		15WHFT1/15WHFT2/ 16WHFT1/16WHFT2/ 16JZFT2	
uqA09.1	A09	39.95	25.61-39.47	15WH-17D/15WH-C/16WH-C			Wu et al., 2013; Wei et al., 2014; Zhang et al., 2019
uqA09.2	A09	42.87	38.52-47.21			15WHFT1/15WHFT2	
uqC02	C02	8.64	7.35-9.92	16WH-14D/16WH-C	15JZDI2/16JZDI2	16ZYFT1/16ZYFT2/ 16WHFT1/16WHFT2/ 16JZFT2	Wei et al., 2014; Wu et al., 2019; Zhang et al., 2019
uqC03.1	C03	22.39	14.76-30.01	15WH-C/16WH-14D	16JZDI1		Wu et al., 2019
uqC03.2	C03	130.79	124.26-137.33		15JZDI2/16JZDI2		

Chr, chromosome; SR, stem resistance; DI, disease index; FT, flowering time.

### Feasible strategies for improving SSR resistance of parents

**Figure 4:**
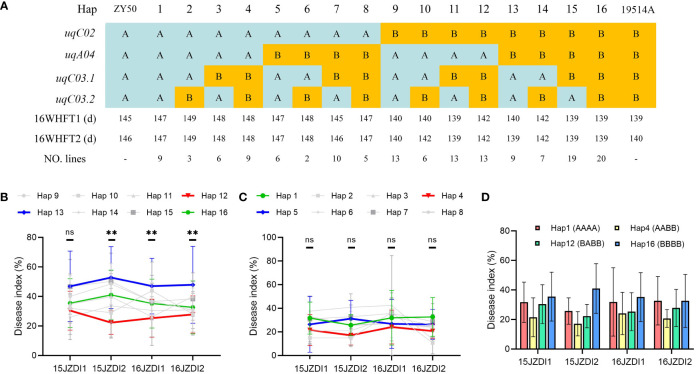
Haplotype analysis in the double haploid (DH) population. **(A)** DH population was divided into 16 haplotypes based on the genotypes of the four quantitative trait loci (QTLs). The number of lines per haplotype and average flowering time (FT) are shown. **(B)** Mean value of disease index **(DI)** and order of haplotypes 9–16 in four replicates. Data are shown as the mean ± SD. Differences between haplotype 12 and haplotype 13 were analyzed for significance; ns indicates no significant difference, **P < 0.01 (one-way analysis of variance). **(C)** Mean value of DI and order of haplotypes 1–8 in four replicates, with the data shown as the mean ± SD. Differences between haplotype 4 and haplotype 5 were analyzed for significance; ns indicates no significant difference (one-way analysis of variance). **(D)** DI values of haplotype 4 were less than those of haplotype 1 in all four replicates; the DI values of haplotype 12 were less than those of haplotype 16 in all four replicates. Data are shown as the mean ± SD.

To explore the application value of the improved SSR resistance of *uqC02*, *uqA04*, *uqC03.1*, and *uqC03.2*, we conducted haplotype analysis of DI in the DH population. Four QTLs divided the 151 DH lines into 16 haplotypes (**Figure 4A**). The obvious differences in the FTs of haplotype 1–8 and haplotype 9–16 suggest that the regulation of SSR resistance of the other three QTLs should be considered based on *uqC02*. When the genotype of *uqC02* was consistent with that of 19514A, haplotype 13 showed the highest DI in all four replicates, whereas its complementary haplotype 12 exhibited a ubiquitously low DI. The difference between the two haplotypes was very significant in three replicates. Additionally, the DI of haplotype 16, which was consistent with that of 19514A, was always between those of haplotype 13 and haplotype 12 (**Figure 4B**), possibly because of the source of additive effects. Interestingly, when the genotype of *uqC02* was consistent with that of ZY50, the above patterns were not significant (**Figure 4C**). We further compared haplotype 1 and haplotype 16 with the other haplotypes. The results showed that in all four replicates, the DI of haplotype 4 was lower than that of haplotype 1 with a difference of 7.8–12%, and the DI of haplotype 12 was smaller than that of haplotype 16, with a difference of 4.8–18.7% (**Figure 4D**). The above results confirm that *uqA04*, *uqC03.1*, and *uqC03.2* regulate SSR resistance, and that introduction of *uqC03.1* and *uqC03.2* from 19514A into ZY50 and *uqA04* from ZY50 into 19514A can enhance SSR resistance.

## Discussion

With the rapid development of genotype analysis technology, phenotypes have become key factors in exploring valuable genetic loci. Using traditional methods, it was difficult to accurately identify the phenotype of SSR resistance in rapeseed, and the identifiable phenotype in the seedling stage did not always accurately reflect the final resistance level. Additionally, the phenotypes of plants cultivated in a greenhouse frequently do not reflect the phenotypes of those in the field, whose development is typically unstable because of the influence of environmental factors (Ding et al., 2021).

Most methods for stem inoculation used in previous studies involved measuring the lesion length within 7 days after inoculation (Zhao and Meng, 2003; Wei et al., 2014; Wei et al., 2016; Wu et al., 2016b; Wang et al., 2018; Wu et al., 2019; Zhang et al., 2019; Ding et al., 2020). In this study, we measured the lesion length a second time at 14 days after inoculation. Compared with the data at 7 dpi, the phenotypic values of the DH population at 14 dpi showed a wider range, the data showed better reproducibility, and more QTL for SSR resistance were identified. In addition, the increase in lesion length from 7 to 14 days was very reproducible after two years, better reflecting the resistance level of materials to SSR after successful infection. These results suggest that appropriately prolonging the inoculation time can reflect the differences in the materials’ resistance levels, and that measuring the lesion length after 14 days of inoculation or the increase in lesion length from 7 to 14 days as the phenotypic value may more accurately reveal their resistance levels. Similar systematic problems were observed in many recent studies, and the infection times were appropriately extended (Zhang et al., 2019; Gupta et al., 2020).

Another method for identifying disease resistance used in this study is natural infection experiments. To increase pathogenic pressure, a 5% suspension of *S. sclerotiorum* hyphae was sprayed onto rapeseed plants at the full-flowering stage. The phenotypic data of four replicates in two years were significantly positively correlated, and QTL related to SSR resistance were repeatedly detected in multiple environments. These results demonstrate that the natural infection experiment is a relatively stable method for detecting SSR resistance; this method was also recently applied in numerous studies (Zhang et al., 2019).

Using the methods described above, we identified 13 SR QTLs, 9 DI QTLs, and 20 FT QTLs. Among them, *uqC02* was detected in all three traits, and is considered as a locus that simultaneously regulates SSR resistance and FT, which is consistent with the results of many previous reports (Wei et al., 2014; Wu et al., 2019; Zhang et al., 2019). Interestingly, *uqC02* was a major QTL that explained more than 40% of the phenotypic variation in the FT in WH and 27.6% of that in the FT in JL, showing a significantly stronger effect than those of other loci. However, *uqC02* only explained approximately 10% of the phenotypic variation in SSR resistance, which is comparable to the effect of other loci, and in this instance cannot be considered as a major QTL. This result suggests that the SSR resistance phenotype in this DH population is not completely controlled by the FT, and the application of other SSR QTL may improve SSR resistance without altering the FT. Notably, the gene regulating the FT in *uqC02* may have been cloned, and *BnaFLC.C2* is considered to have caused the variation in FT (Chen et al., 2018). Based on the close relationship between the FT and SSR resistance, *BnaFLC.C2* likely also regulates SSR resistance in *uqC02*. Although *uqC02* has some application value in improving the rapeseed growth period (Fang et al., 2021), its application value in SSR resistance improvement should be further investigated because of the link between the FT and SSR resistance.

*uqA09.1* and *uqA09.2* were predicted to regulate SR and FT, respectively, but the confidence interval of the two loci showed some overlap, and a single locus may control SSR resistance and FT simultaneously. Previous studies also demonstrated that these loci are responsible for SSR resistance (Wu et al., 2013; Wei et al., 2014; Zhang et al., 2019); however, because the interval is also an area of concentrated rapeseed yield genes (Liu et al., 2015; Shi et al., 2019), a more specific and elaborate design is required before this segment can be used to improve SSR resistance.

Regulation of the FT by *uqA07* was stably detected in the winter rapeseed environment but not in the spring rapeseed environment, indicating that *uqA07* is an environment-specific QTL for FT. As *uqA07* was involved in regulating SR but did not affect NI, it was not further analyzed nor applied in this study. However, *uqA07* may play an important role in finely regulating the FT in rapeseed, and *BnaFT.A07* may be the functional gene of *uqA07*.

Several other SSR resistance QTLs were also identified in this study, among which the resistance source of *uqA04* was ZY50 and that of *uqC03.1* and *uqC03.2* was 19514A. The phenotypic contribution and additive effect of the three QTLs were similar, and thus they were considered to have the same resistance levels. Previous studies reported *uqC03.1* as a SSR resistance QTL (Wu et al., 2019), whereas *uqA04* and *uqC03.2* were previously unreported. In this study, we confirmed that materials with significant differences in SSR resistance can be obtained by using a reasonable combination of these three loci. According to the source of resistance, application of *uqA04* may effectively improve the SSR resistance of the backbone parent 19514A. Our results confirm that low SSR resistance materials can also provide SSR resistance sources; we also demonstrated that the polymerization of SSR resistance loci in low SSR resistance materials is another effective strategy for improving rapeseed SSR resistance. These results can be applied when selecting SSR resistance cultivars in rapeseed. In addition, our results provide a foundation for further studies of major diseases in other crops and a reference for evaluating crop diseases.

## Data availability statement

The original contributions presented in the study are included in the article/**Supplementary Materials**. Further inquiries can be directed to the corresponding authors.

## Author contributions

XHZ conducted most experiments and wrote the original draft. XL, HL, and RX participated in phenotypic data collection. PW and XMZ participated in data analysis. ZW, LW, and JH designed the experiments and were involved in reviewing and editing the manuscript. DH and GY supervised the project. All authors read and contributed to the revision of manuscript.

## Funding

This research was supported by the Program for Modern Agricultural Industrial Technology System (CARS-12), Open Fund of the National Key Laboratory of Crop Genetic Improvement (ZK201909), and scientific research start funds of Hainan University.

## Acknowledgments

We would like to thank Editage (www.editage.cn) for English language editing.

## Conflict of interest

The authors declare that the research was conducted in the absence of any commercial or financial relationships that could be construed as a potential conflict of interest.

## Publisher’s note

All claims expressed in this article are solely those of the authors and do not necessarily represent those of their affiliated organizations, or those of the publisher, the editors and the reviewers. Any product that may be evaluated in this article, or claim that may be made by its manufacturer, is not guaranteed or endorsed by the publisher.

## Supplementary material

Supplementary Figure 1 Distribution of the flowering time (FT) of double haploid (DH) populations in ten replicates.
