# Supplementary figures and images for: Quantitative trait locus mapping and improved resistance to sclerotinia stem rot in a backbone parent of rapeseed (Brassica napus L.)
Source: Front Plant Sci. 2022 Nov 10;13:1056206. doi: 10.3389/fpls.2022.1056206 (PMC9684713; doi:10.3389/fpls.2022.1056206)

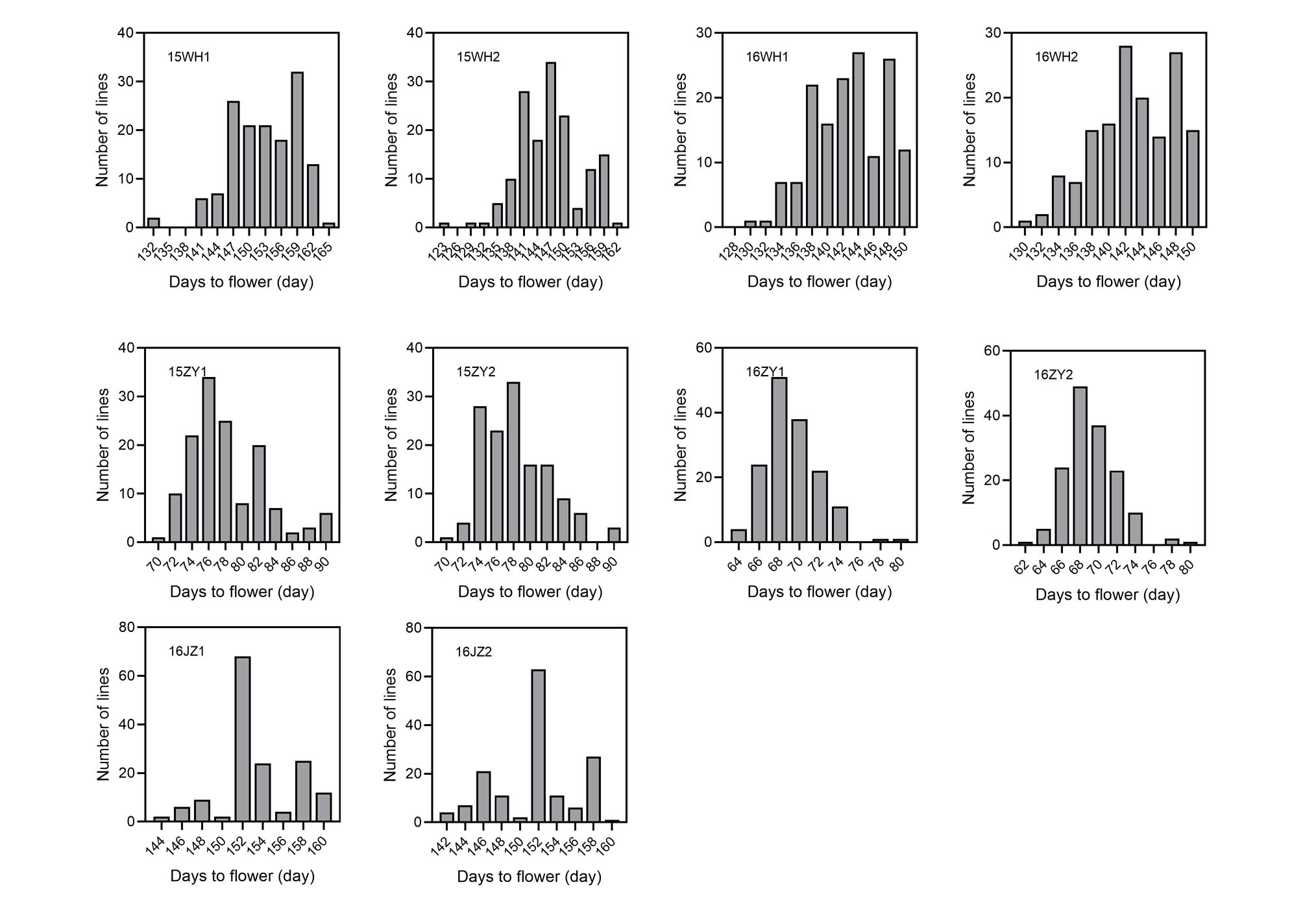

Supplement: Supplementary Figure 1 — Distribution of the flowering time (FT) of double haploid (DH) populations in ten replicates. [file Image_1.jpeg]
