# Supplementary material for: Quantitative trait locus mapping and improved resistance to sclerotinia stem rot in a backbone parent of rapeseed (Brassica napus L.)
Source: Front Plant Sci. 2022 Nov 10;13:1056206. doi: 10.3389/fpls.2022.1056206 (PMC9684713; doi:10.3389/fpls.2022.1056206)
Supplement: Supplementary file 2 [file Table_1.docx]

**SUPPLEMENTARY TABLE 1 correlation analysis of stem resistance (SR).**

|  | 15WHSR-7D | 15WHSR-17D | 15WHSR-C | 16WHSR-7D | 16WHSR-14D | 16WHSR-C |
| --- | --- | --- | --- | --- | --- | --- |
| 15WHSR-7D | 1.000 | 1.487e-028 | 1.230e-006 | 0.118 | 0.017 | 0.029 |
| 15WHSR-17D | 0.795^**^ | 1.000 | 1.882e-042 | 0.107 | 0.002 | 0.002 |
| 15WHSR-C | 0.420^**^ | 0.884^**^ | 1.000 | 0.406 | 0.039 | 0.018 |
| 16WHSR-7D | 0.131 | 0.147 | 0.076 | 1.000 | 8.682e-033 | 3.130e-004 |
| 16WHSR-14D | 0.199^*^ | 0.274^**^ | 0.188 | 0.789^**^ | 1.000 | 5.866e-037 |
| 16WHSR-C | 0.183^*^ | 0.281^**^ | 0.215^*^ | 0.293^**^ | 0.818^**^ | 1.000 |

The lower left part is the correlation coefficient, and the upper right part is the P-value, **P < 0.01 (Pearson correlation coefficient).
