# Supplementary material for: Quantitative trait locus mapping and improved resistance to sclerotinia stem rot in a backbone parent of rapeseed (Brassica napus L.)
Source: Front Plant Sci. 2022 Nov 10;13:1056206. doi: 10.3389/fpls.2022.1056206 (PMC9684713; doi:10.3389/fpls.2022.1056206)
Supplement: Supplementary file 3 [file Table_2.docx]

**SUPPLEMENTARY TABLE 2 Quantitative trait loci (QTL) information of stem resistance (SR).**

| **Traits** | **QTL** | **Chr** | **LOD** | **PVE (%)** | **Add** | **CI (cM)** | **Peak (cM)** |
| --- | --- | --- | --- | --- | --- | --- | --- |
| 15WHSR-7D | *qSRC02-1* | C02 | 4.29 | 10.8 | -0.88 | 31.1-42.8 | 41.5 |
| 15WHSR-17D | *qSRA07-1* | A07 | 3.33 | 8.1 | -1.81 | 0-16.1 | 6.50 |
|  | *qSRA09-1* | A09 | 3.59 | 8.7 | 1.92 | 17.9-43.8 | 34.5 |
| 15WHSR-C | *qSRA07-1* | A07 | 4.11 | 10.0 | -1.09 | 0-15.9 | 6.50 |
|  | *qSRA09-1* | A09 | 4.08 | 10.5 | 1.18 | 16.1-46.5 | 34.3 |
|  | *qSRC03-1* | C03 | 3.16 | 7.5 | 1.63 | 10.8-30.9 | 25.4 |
| 16WHSR-7D | *qSRA03-1* | A03 | 2.9 | 6.1 | 0.62 | 6.0-10.2 | 7.7 |
| 16WHSR-14D | *qSRA08-1* | A08 | 3.85 | 8.2 | -1.78 | 44.4-44.7 | 45.2 |
|  | *qSRC02-2* | C02 | 3.65 | 6.5 | -0.95 | 0-18.1 | 9.5 |
|  | *qSRC03-1* | C03 | 2.65 | 4.3 | 0.76 | 3.5-41.1 | 35.2 |
| 16WHSR-C | *qSRA07-2* | A07 | 2.91 | 5.4 | 0.43 | 10.8-29.3 | 18.9 |
|  | *qSRA09-1* | A09 | 2.90 | 5.4 | -0.46 | 11.9-31.4 | 30.7 |
|  | *qSRC02-2* | C02 | 4.84 | 11.2 | -0.65 | 0-10.5 | 9.5 |

QTL were designated using the initials of ‘q’ and the abbreviate of the trait, chromosome name, and a ‘-’ followed by a number distinguishing from others in the same chromosome.
