# Supplementary material for: Quantitative trait locus mapping and improved resistance to sclerotinia stem rot in a backbone parent of rapeseed (Brassica napus L.)
Source: Front Plant Sci. 2022 Nov 10;13:1056206. doi: 10.3389/fpls.2022.1056206 (PMC9684713; doi:10.3389/fpls.2022.1056206)
Supplement: Supplementary file 4 [file Table_3.docx]

**SUPPLEMENTARY TABLE 3 Correlation analysis of disease index (DI).**

|  | 15JZDI1 | 15JZDI2 | 16JZDI1 | 16JZDI2 |
| --- | --- | --- | --- | --- |
| 15JZDI1 | 1 | 6.998e-018 | 0.022 | 5.680e-007 |
| 15JZDI2 | 0.6588^**^ | 1 | 1.921e-004 | 3.760e-012 |
| 16JZDI1 | 0.200^*^ | 0.304^**^ | 1 | 0.002 |
| 16JZDI2 | 0.418^**^ | 0.531^**^ | 0.246^**^ | 1 |

The lower left part is the correlation coefficient, and the upper right part is the P-value, **P < 0.01 (Pearson correlation coefficient).
