# Supplementary material for: Quantitative trait locus mapping and improved resistance to sclerotinia stem rot in a backbone parent of rapeseed (Brassica napus L.)
Source: Front Plant Sci. 2022 Nov 10;13:1056206. doi: 10.3389/fpls.2022.1056206 (PMC9684713; doi:10.3389/fpls.2022.1056206)
Supplement: Supplementary file 5 [file Table_4.docx]

**SUPPLEMENTARY TABLE 4 Quantitative trait loci (QTL) information of disease index (DI).**

| **Traits** | **QTL** | **Chr** | **LOD** | **PVE(%)** | **Add** | **CI (cM)** | **Peak (cM)** |
| --- | --- | --- | --- | --- | --- | --- | --- |
| 15JZDI2 | *qDIA04-1* | A04 | 4.42 | 8.5 | -5.66 | 0-12.2 | 4.7 |
|  | *qDIC02-1* | C02 | 5.82 | 12.2 | -7.16 | 0.9-16.8 | 9.4 |
|  | *qDIC03-2* | C03 | 5.49 | 10.7 | 6.28 | 120.7-137.0 | 131.9 |
| 16JZDI1 | *qDIA03-2* | A03 | 4.04 | 9.6 | -5.42 | 56.6-72.9 | 65.5 |
|  | *qDIA04-1* | A04 | 3.61 | 8.5 | -5.27 | 0-21.8 | 11.3 |
|  | *qDIC03-1* | C03 | 3.57 | 8.6 | 5.39 | 4.5-27.9 | 18.3 |
|  | *qDIC08-1* | C08 | 3.33 | 7.8 | 6.42 | 60.3-80.6 | 73.4 |
| 16JZDI2 | *qDIC02-1* | C02 | 3.82 | 8.6 | -4.89 | 0-17.1 | 9.2 |
|  | *qDIC03-2* | C03 | 2.54 | 5.3 | 3.61 | 118.1-140.0 | 128.8 |

QTL were designated using the initials of ‘q’ and the abbreviate of the trait, chromosome name, and a ‘-’ followed by a number distinguishing from others in the same chromosome.
