# Supplementary material for: Quantitative trait locus mapping and improved resistance to sclerotinia stem rot in a backbone parent of rapeseed (Brassica napus L.)
Source: Front Plant Sci. 2022 Nov 10;13:1056206. doi: 10.3389/fpls.2022.1056206 (PMC9684713; doi:10.3389/fpls.2022.1056206)
Supplement: Supplementary file 6 [file Table_5.docx]

**SUPPLEMENTARY TABLE 5 Correlation analysis of flowering time (FT).**

|  | 15ZYFT1 | 15ZYFT2 | 16ZYFT2 | 16ZYFT1 | 15WHFT1 | 15WHFT2 | 16WHFT1 | 16WHFT2 | 16JZFT1 | 16JZFT2 |
| --- | --- | --- | --- | --- | --- | --- | --- | --- | --- | --- |
| 15ZYFT1 | - | 1.163e-039 | 3.147e-019 | 4.232e-021 | 1.299e-007 | 1.011e-020 | 3.693e-019 | 1.803e-018 | 3.709e-011 | 2.694e-018 |
| 15ZYFT2 | 0.850^**^ | - | 2.548e-014 | 1.882e-014 | 4.749e-008 | 1.812e-020 | 3.034e-020 | 1.101e-018 | 1.388e-010 | 2.616e-013 |
| 16ZYFT2 | 0.665^**^ | 0.589^**^ | - | 1.938e-027 | 4.897e-006 | 3.866e-013 | 2.327e-016 | 2.267e-014 | 4.162e-009 | 8.448e-012 |
| 16ZYFT1 | 0.690^**^ | 0.591^**^ | 0.735^**^ | - | 2.190e-005 | 7.679e-014 | 3.150e-015 | 6.302e-014 | 9.909e-008 | 1.058e-009 |
| 15WHFT1 | 0.437^**^ | 0.449^**^ | 0.368^**^ | 0.343^**^ | - | 4.021e-015 | 7.931e-014 | 2.057e-016 | 9.415e-010 | 1.256e-010 |
| 15WHFT2 | 0.685^**^ | 0.680^**^ | 0.545^**^ | 0.558^**^ | 0.589^**^ | - | 2.056e-043 | 3.076e-048 | 2.482e-015 | 1.452e-024 |
| 16WHFT1 | 0.664^**^ | 0.677^**^ | 0.602^**^ | 0.583^**^ | 0.566^**^ | 0.846^**^ | - | 8.314e-063 | 1.908e-019 | 2.103e-030 |
| 16WHFT2 | 0.654^**^ | 0.655^**^ | 0.568^**^ | 0.560^**^ | 0.612^**^ | 0.869^**^ | 0.918^**^ | - | 9.235e-018 | 3.871e-030 |
| 16JZFT1 | 0.527^**^ | 0.512^**^ | 0.456^**^ | 0.417^**^ | 0.479^**^ | 0.585^**^ | 0.644^**^ | 0.619^**^ | - | 6.734e-016 |
| 16JZFT2 | 0.651^**^ | 0.569^**^ | 0.518^**^ | 0.469^**^ | 0.499^**^ | 0.705^**^ | 0.760^**^ | 0.758^**^ | 0.594^**^ | - |

The lower left part is the correlation coefficient, and the upper right part is the P-value, **P < 0.01 (Pearson correlation coefficient).
