# Supplementary material for: Quantitative trait locus mapping and improved resistance to sclerotinia stem rot in a backbone parent of rapeseed (Brassica napus L.)
Source: Front Plant Sci. 2022 Nov 10;13:1056206. doi: 10.3389/fpls.2022.1056206 (PMC9684713; doi:10.3389/fpls.2022.1056206)
Supplement: Supplementary file 7 [file Table_6.docx]

**SUPPLEMENTARY TABLE 6 Quantitative trait loci (QTL) information of flowering time (FT).**

| **Traits** | **QTL** | **Chr** | **LOD** | **PVE (%)** | **Add** | **Int(cM)** | **Peak(cM)** |
| --- | --- | --- | --- | --- | --- | --- | --- |
| 15ZYFT1 | *qFTA02-1* | A02 | 2.84 | 7.0 | 6.62 | 44.0-63.0 | 52.2 |
| 16ZYFT1 | *qFTC02-1* | C02 | 3.04 | 8.1 | 1.85 | 0-8.9 | 0 |
| 16ZYFT2 | *qFTC02-1* | C02 | 2.53 | 6.2 | 1.65 | 0-9.9 | 6.1 |
| 15WHFT1 | *qFTA07-1* | A07 | 4.02 | 10.2 | -10.06 | 0-10.1 | 1.7 |
| 15WHFT3 | *qFTA05-1* | A05 | 3.54 | 4.5 | 1.49 | 44.8-88.7 | 58.9 |
|  | *qFTA07-1* | A07 | 2.77 | 3.3 | -1.32 | 0-10.4 | 0.0 |
|  | *qFTC02-1* | C02 | 26.03 | 48.2 | 5.27 | 3.4-10.4 | 7.1 |
|  | *qFTC03-1* | C03 | 3.51 | 4.3 | -1.45 | 94.7-113.8 | 104.7 |
| 16WHFT1 | *qFTA02-2* | A02 | 3.02 | 3.3 | -0.87 | 7.2-28.8 | 18.1 |
|  | *qFTA07-1* | A07 | 4.38 | 4.9 | -1.09 | 0-16.3 | 9.5 |
|  | *qFTA09-1* | A09 | 3.52 | 3.8 | 0.96 | 36.2-48.8 | 45.7 |
|  | *qFTA10-1* | A10 | 4.35 | 4.8 | 1.05 | 54.9-72.1 | 63.1 |
|  | *qFTC02-1* | C02 | 26.84 | 45.8 | 3.48 | 4.4-10.6 | 8.2 |
| 16WHFT2 | *qFTA07-1* | A07 | 4.28 | 4.7 | -1.11 | 0.5-20.3 | 11.0 |
|  | *qFTA09-1* | A09 | 4.75 | 5.3 | 1.19 | 35.8-47.8 | 40.3 |
|  | *qFTC02-1* | C02 | 27.07 | 43.8 | 3.6 | 5.9-10.7 | 9.3 |
|  | *qFTC02-2* | C02 | 2.98 | 3.3 | -0.95 | 44.7-65.3 | 53.2 |
| 16JZFT2 | *qFTA04-1* | A04 | 2.90 | 4.6 | 0.92 | 0-10.4 | 2.2 |
|  | *qFTA07-1* | A07 | 3.10 | 4.5 | -0.93 | 0-18.6 | 3.9 |
|  | *qFTC02-1* | C02 | 15.39 | 27.6 | 2.44 | 5.5-11.1 | 9.4 |

QTL were designated using the initials of ‘q’ and the abbreviate of the trait, chromosome name, and a ‘-’ followed by a number distinguishing from others in the same chromosome.
